# Cerebrolysin Attenuates Heat Shock Protein (HSP 72 KD) Expression in the Rat Spinal Cord Following Morphine Dependence and Withdrawal: Possible New Therapy for Pain Management

**DOI:** 10.2174/157015911795017100

**Published:** 2011-03

**Authors:** Hari S Sharma, Syed F Ali, Ranjana Patnaik, Sibilla Zimmermann-Meinzingen, Aruna Sharma, Dafin F Muresanu

**Affiliations:** 1 Laboratory of Cerebrovascular Research, Department of Surgical Sciences, Anesthesiology & Intensive Care Medicine, University Hospital, Uppsala University, SE-75185 Uppsala Sweden; 2 Neurochemistry Laboratory, Division of Neurotoxicology, USFDA/National Center for Toxicological Research, Jefferson, AR, USA; 3 Department of Biomaterials, School of Biomedical Engineering, Institute of Technology, Banaras Hindu University, Varanasi, India; 4 Ever NeuroPharma, Unterach, Austria; 5 Department of Neurology, University of Medicine and Pharmacy, Cluj-Napoca, Romania

**Keywords:** Morphine, heat shock proteins (HSP 72 kD), morphine tolerance, withdrawal symptoms, stress reaction, cerebrolysin, growth factors, pain perception, analgesia.

## Abstract

The possibility that pain perception and processing in the CNS results in cellular stress and may influence heat shock protein (HSP) expression was examined in a rat model of morphine dependence and withdrawal. Since activation of pain pathways result in exhaustion of growth factors, we examined the influence of cerebrolysin, a mixture of potent growth factors (BDNF, GDNF, NGF, CNTF etc,) on morphine induced HSP expression. Rats were administered morphine (10 mg/kg, s.c. /day) for 12 days and the spontaneous withdrawal symptoms were developed by cessation of the drug administration on day 13^th^ that were prominent on day 14^th^ and continued up to day 15^th^ (24 to 72 h periods). In a separate group of rats, cerebrolysin was infused intravenously (5 ml/kg) once daily from day one until day 15^th^. In these animals, morphine dependence and withdrawal along with HSP immunoreactivity was examined using standard protocol. In untreated group mild HSP immunoreaction was observed during morphine tolerance, whereas massive upregulation of HSP was seen in CNS during withdrawal phase that correlated well with the withdrawal symptoms and neuronal damage. Pretreatment with cerebrolysin did not affect morphine tolerance but reduced the HSP expression during this phase. Furthermore, cerebrolysin reduced the withdrawal symptoms on day 14^th^ to 15^th^. Taken together these observations suggest that cellular stress plays an important role in morphine induced pain pathology and exogenous supplement of growth factors, i.e. cerebrolysin attenuates HSP expression in the CNS and induce neuroprotection. This indicates a new therapeutic role of cerebrolysin in the pathophysiology of drugs of abuse, not reported earlier.

## INTRODUCTION

Pain perception and its regulation within the central nervous system (CNS) is complex and the cellular or molecular mechanisms of pain processing are still not well known [1-3]. Previous experiments from our laboratory suggest that the micro-fluid environment of the brain and spinal cord plays important role in neuropathic pain [1, 2]. Thus, peripheral nerve lesion or ligation is associated with breakdown of the blood-spinal cord barrier (BSCB) to serum albumin in several segments of the corresponding spinal cord within 1 to 10 weeks after the primary insult [1, 2]. In addition, the areas around the albumin leakage showed distinct morphological alterations in the neurons and the glial cells [1]. This suggests that alterations in the fluid microenvironment of the CNS during pain processing and perception could be instrumental in causing structural changes in the spinal cord and may lead to an early neurodegeneration [1-4]. Therefore, in order to enhance patient care, new therapeutic measures are needed to prevent these neurodegenerative changes in the CNS that are often associated with alterations in pain response or pain pathways.

Morphine dependence and withdrawal result in marked alterations in pain perception and/or modulation of pain pathways [5, 6]. However, the mechanisms of morphine dependence or withdrawal induced structural and functional changes in the spinal cord are still not well characterized [7]. Previous reports from our laboratory show that morphine dependence and withdrawal in rats are often associated with alterations in the BBB function [8-11]. Furthermore, spontaneous withdrawal by cessation of morphine administration resulted in selective and specific neuronal and glial cell damage in the cerebral cortex, hippocampus, brainstem, thalamus and hypothalamus [8-12]. These changes were most pronounced in the brain within 48 to 72 h after the discontinuation of morphine injection [8-10, 12]. The probable mechanisms of this selective neurodegenerative changes during morphine withdrawal is still unclear. However, observed a profound increase in heat shock protein 72 kD (HSP) expression in the areas showing neuronal distortion and damage [9, 11, 12]. This indicates that cellular stress caused by morphine withdrawal may play important determining role in the BBB dysfunction [13-18] and subsequently neuronal and glial damage in the brain [19-24]. It is therefore possible that drugs or therapeutic agents that are able to reduce the stress response caused by morphine dependence or withdrawal could be able to attenuate these neurodegenerative changes.

There are reasons to believe that several neurotrophic factors are actively involved in the process of psychostimulant induced dependence and withdrawal responses within the CNS [7, 23-27]. Thus, infusion of brain derived neurotrophic factor (BDNF) or neurotensin-4 (NT-4) into the ventral tegmental area (VTA) of the midbrain attenuated morphine or cocaine induced changes in the neuronal morphology and dendritic structures in animal models [25, 28]. But the local infusion of nerve growth factor (NGF) failed to affect morphine or cocaine induced changes in the VTA neurons [29]. On the contrary, intracerebral infusion of neurotensin-3 (NT-3) into the VTA potentiated the behavioral effects of cocaine [30]. Moreover, intra-VTA infusion of a neutralizing antibody for NT-3 also enhanced the cocaine induced behavioral responses [31]. These observations suggest that the role of specific neurotrophic factors in the brain during drugs of abuse are still unclear and require further investigations. Since morphine, cocaine, amphetamine and other psychostimulants affects both neuronal and glial cell response within the brain [12-16, 32], a combination of BDNF, glial cell derived neurotrophic factors (GDNF) and other growth factors may be able to prevent or reduce the cellular and molecular responses of the brain following substance abuse.

Previous studies in our laboratory demonstrated that cerebrolysin, a combination of several neurotrophic factors (BDNF, GDNF, NGF, CNTF etc,) [33, 34] is able to reduce HSP 72 KD response and brain damage following heat stress in rats [35]. This suggests that a suitable combination of neurotrophic factors may reduce cellular stress to induce neuroprotection [36-38]. Thus, the possibility that cerebrolysin could influence morphine dependence and withdrawal induced HSP expression and cellular changes in the brain was examined in this investigation in our rat model of morphine dependence and withdrawal. 

## MATERIALS AND METHODS

### Animals

Experiments were carried out on Charles Foster male rats (200-250 g body weight) housed at controlled room temperature 21±1° C with 12 h light and 12 h dark schedule. Food and tap water were supplied *ad libitum* before the experiments. All experiments were conducted according to National Institute of Health (NIH) guidelines for care and handling of experimental animals and approved by local Institutional Ethics Committee.

### Morphine Dependence and Withdrawal

Morphine Sulfate (7, 8-Dihydro-4,5α-epoxy-17-methylmorphinan-3,6-α-diol sulfate; Cat. Nr. M8777, Sigma-Aldrich, Inc, Natick, MA, USA, Mol. Wt. 668.76, anhydrous) was dissolved freshly every day in sterile saline (30 mg/ml) 5-10 min before its administration in rats [11,12]. Each rat was administered 10 mg/kg Morphine subcutaneously once daily for 12 days to develop dependence [11]. Spontaneous withdrawal was induced by abrupt cessation of morphine injection on day 13 and animals were monitored for 72 h after morphine withdrawal [12].

### Cerebrolysin Treatment

In another group of rats, cerebrolysin (Ebewe NeuroPharma, Unterach, Austria) was administered intravenously. For this purpose, a polythene cannula (PE 25) was implanted into the right jugular vein aseptically 4 days before commencement of the experiments [39]. The cannula was flushed regularly with heparinized saline to keep it patent [40]. Cerebrolysin (5 ml/kg) was infused through the cannula slowly (ca. 0.3 ml/min) daily once before the administration of morphine and continued even after cessation of morphine injection (from day 1 to day 15), i.e., up to 72 h after the withdrawal. This dose of cerebrolysin is known to induce neuroprotection in hyperthermia and/or brain injuries [33, 35]. 

### Parameters Measured

The following parameters were measured in cerebrolysin treated or untreated groups subjected to morphine dependence and withdrawal.

### Morphine Analgesia

Morphine analgesia was evaluated using latency of tail-flick response to radiant heat as nociceptive stimulus [11]. The animals were placed in individual Perspex restraint boxes (18x5x6 cm) having several round holes on their walls for free ventilation and the entire length of the tail remained outside the box [41]. The test was carried out by placing the middle part of the tail (keratinised scales were already removed 24 h before) on an electrically heated tungsten wire (50 °C). The cut-off latency for tail-flick response was kept to 15 sec (see Table **2**).

The control latency of tail-flick response in each rat was determined before morphine administration. Following morphine administration, the analgesic tests were carried out at intervals of 10 to 15 min for 120 min. The latency response for each test was determined on the basis of average of three successive readings of the tail-flick response. The actual increase in latency response induced by morphine was calculated by deducting the control latency value from that observed following morphine administration. The maximum percent response (MPR) was calculated using the formula [41, 42] as follows: 

% MPR = Test latency - Control latency ÷ cut-off time - control latency x 100 [41].

The development of tolerance to analgesic response of morphine in rats occurred with single daily injection of morphine (10 mg/kg, i.p.). The onset of development of tolerance was found to commence from the 4th day onwards and full tolerance developed by the 12th day of morphine treatment [12].

### Morphine Withdrawal Symptoms

The withdrawal symptoms were studied after the development of complete tolerance to morphine analgesia. From our studies it was found that 12 days are needed to develop morphine tolerance [22]. To induce spontaneous morphine withdrawal symptoms, the morphine administration was withdrawn on the 13^th^ day and the symptoms were noted daily for 72 h. To evaluate the spontaneous morphine withdrawal symptoms, the rats were removed from their individual cages and placed in new cages. Each rat was observed for 30 min and the gross abnormal behavior, if any, were carefully recorded as previously demonstrated [43, 44] and as described below.

#### (a) Wet-Shakes

These are vigorous shaking movements of the head and/or body of the rat similar to those produced by an animal when wet with water. The number of shakes during the 30 min observation period was recorded.

#### (b) Piloerection

This is the condition in which the fur on the body surface stood erect. The presence or absence of piloerection in 30 min of observation period was noted.

#### (c) Writhing

This syndrome consists of dragging of the abdomen along the floor of the cage with “sucking in" the abdominal wall or stretching and arching of the back, neither of which was accompanied with yawning. The presence or absence of this syndrome during 30 min observation period was recorded.

#### (d) Teeth Chattering 

This is an audible distinct sound and was identical to the gnawing sound produced by a rat while eating food pellets. Within the 30 min observation period, the presence or absence of such sound was noted.

#### (e) Diarrhoea 

The presence or absence of formless stool generally adhered to the base of the tail was noted during 30 min observation period.

#### (f) Aggressive Behavior

The aggressive behavior of the animal was observed by placing an untreated rat in the cage of the morphine-withdrawn rats for 5 min. Immediately after placement, the animals started fighting with squeaking and biting attempts. Animals often show erect posture and facing each other like a boxing stance. The morphine withdrawn rats exhibiting such symptoms were termed as aggressive.

#### (g) Loss of Body Weight

The body weight of the morphine withdrawn rats was compared with the weight of animals immediately before morphine withdrawal [11, 12]. 

The animals treated once daily morphine (10 mg/kg, i.p.) developed full tolerance on the day 12 as evident with their analgesic response, cataleptic response and hyperthermic response. In these morphine dependent rats, cessation of morphine injection resulted in the appearance of spontaneous withdrawal symptoms within 12 h [10-12]. The withdrawal symptoms were mainly observed to be the loss of body weight, "wet shake" phenomena, piloerection, writhing, teeth chattering, diarrhea and aggressive and jumping behavior [8-12]. These withdrawal symptoms continue to worsen with time. Thus, the withdrawal symptoms were aggravated at 24 and 48 h after the cessation of morphine. No apparent reduction in the withdrawal symptoms was observed until 72 h later after the cessation of morphine administration [7-10, 45]. 

### Physiological Variables

The mean arterial blood pressure (MABP), heart rate, arterial pH and blood gases were examined in controls, morphine treated and dependent rats as well as in rats subjected to morphine withdrawal according to standard procedures [39, 40].

### Stress Symptoms

In morphine withdrawal rats, the stress symptoms were most severe following 24 and 48 h after the cessation of morphine administration. These symptoms include jumping, teeth chattering, writhing, circular motion and restlessness in the cages as evident with hyper-locomotor activity. The occurrence of diarrhea was most prominent on the 2nd day of morphine withdrawal. At post-mortem, many microhemorrhages were noted in the mucosal wall of the stomach indicating formation of stress-ulcers [9-12].

### Morphine Induced Changes in Body Temperature

Changes in the core body temperature following morphine administration was recorded using a thermistor probe (Yellow Spring Co., USA) inserted through the rectum up to a length of 6 cm and it was held in place by wrapping an adhesive leucoplast around the base of the tail [18,19]. The probe was connected to a 6-channel telethermometer (Electromed, UK) that was powered through a voltage stabilizer. The calibration of the apparatus was checked every time before the start of the experiment [18-20].

### Morphological Investigation

We examined HSP 72 kD immunoreactivity at light microscopy and ultrastructural changes in untreated or cerebrolysin treated rats subjected to morphine dependence or withdrawal protocols as described above [12, 46-48]. For this purpose, at the end of the experiment, animals were anesthetized deeply with Equithesin (3 ml/kg, i.p.) and the chest was opened rapidly, heart was exposed and the right auricle was incised. Immediately, after the incision of the right auricle, a butterfly needle (21 G) connected to perfusion system was inserted onto the left ventricle and the intravascular blood was washed out by perfusing about 50 ml of cold 0.1 M phosphate buffer (pH 7.0) followed by perfusion with cold 150 ml 4 % paraformaldehyde in 0.1 M phosphate buffer as described earlier [46]. After perfusion, the animals were wrapped in an aluminum foil and placed overnight in a refrigerator at 4 °C. The next day, the brain and spinal cord was dissected out and placed in the same fixative for 1 week at 4 °C. After one week of tissue preservation, small pieces of the desired brain or spinal cord area were dissected out and about 40 µm thick vibratome sections were cut and collected in different wells in 0.1 M phosphate buffer for immunostaining as free floating sections [48]. Other pieces of tissues from the identical brain areas were post-fixed in osmium tetraoxide and embedded in plastic (Epon 812) for transmission electron microscopy (see below).

### Heat Shock Protein (72 kD) Immunoreactivity

The HSP expression was examined in the brain and spinal cord using immunohistochemistry employing antibodies directed against HSP-72 kD (Amersham, England; for details see [48, 49]. 

In brief, tissue sections (40 µm thick) were cut on a Vibratome (Oxford Instruments, UK) then transferred to the primary antibody solution (mouse anti-HSP antiserum 1:500) and normal swine serum (1:30 in phosphate buffer saline, PBS) and incubated free floating under agitation for 36 h at room temperature [46,48]. Immuno-complexes were localized by incubating the sections for 6-7 min in a solution containing 75 µg (microgram) of DAB and 30 ml of 30 % H2O2/100 ml of Tris-HCl buffer. The sections were washed in 0.15 M sodium cacodylate buffer and post-fixed for 20 min in 2 % OsO_4_ dissolved in cacodylate buffer. They were then dehydrated in a graded series of ethanol, embedded in Epon between acetate foils and polymerized at 60°C for 48 h [48, 49]. The sections were examined under a light microscope for evaluation of the immunolabelling. For comparison, one section in each group was not osmicated to see the immuno-labelling against a light background.

### Transmission Electron Microscopy

Epon embedded tissue pieces from the untreated or cerebrolysin treated brain or spinal cord were sectioned for high resolution microscopy (about 1 µm thick) and stained with toluidine blue [46,48,49]. The desired areas of the tissue block then further trimmed and ultrathin sections were cut on ultramicrotome (LKB, Sweden) using diamond knife. These ultrathin sections were collected on one-hole grid and some of them were counterstained with lead citrate and uranyl acetate before viewing under a Phillips or Hitachi Transmission Electron Microscope [46]. The EM grids were examined in a blinded fashion for neurovascular relations in cerebrolysin treated animals and compared with untreated group subjected to morphine dependence or withdrawal groups [see 11, 12]. In some group of untreated or cerebrolysin treated animals that were subjected to morphine dependence and withdrawal, Lanthanum chloride (LaCl3; 2.5 % solution) was added to the fixative during perfusion. Since La^3+^ is an electron dense product, it can be visualized at TEM without any further processing [50]. Thus, La^3+^ transport across the microvessels can be easily examined in these groups [51, 52].

## STATISTICAL ANALYSES OF DATA

The quantitative (physiological variables) data were analyzed using ANOVA followed by Dunnet’s test for multiple group comparison using one control group. The semiquantitative data (behavioral and morphological investigations) were analyzed using non-parametric Chi-Square test. A P-value < 0.05 was considered significant.

## RESULTS

### Effect of Cerebrolysin on Morphine Analgesia and Dependence

Cerebrolysin treatment did not affect morphine analgesia or dependence in rats. Thus, untreated group developed morphine dependence from the day 4^th^ and onwards and cerebrolysin administration did not alter this pattern. In cerebrolysin treated animals, morphine administration resulted in gradual loss of analgesia from the 2^nd^ day onwards and on the 4^th^ day of injection achieved quite good morphine dependence (results not shown). On the day 10^th^, complete dependence of morphine was seen in both untreated or cerebrolysin treated animals. No apparent differences in untreated or cerebrolysin treated animals were noted on the 12^th^ day of morphine treatment.

### Effect of Cerebrolysin on Morphine Withdrawal Symptoms

Cerebrolysin treatment markedly reduced the morphine withdrawal symptoms as seen on day 13, (24 h after morphine withdrawal) and onwards (Table **1**). Thus, in cerebrolysin treated group, rats showed milder withdrawal symptoms as compared to untreated rats during this phase of morphine withdrawal (Table **1**). This effect of cerebrolysin was most pronounced on reducing aggressive behavior, teeth chattering, weight loss and body temperature changes (Table **1**). This effect of cerebrolysin was most pronounced on the day 13 and 14 (days 1 and 2 after morphine withdrawal).

### Effect of Cerebrolysin on Morphine Induced Physiological Variables

Cerebrolysin treatment, slightly but significantly attenuated alterations in physiological variables during morphine withdrawal. However, changes in these variables were not affected by cerebrolysin during the phase of morphine dependence (Table **2**). Thus, alterations in heart rate and respiration were markedly reduced in cerebrolysin treated animals after morphine withdrawal (day 13 to 15). The magnitude and intensity of reduction in changes in these variables were most marked on the day 13 in cerebrolysin treated rats after morphine withdrawal (Table **2**). 

### Effect of Cerebrolysin on Morphine Induced HSP Expression

**Fig. (1):**
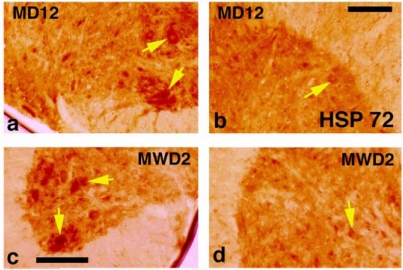
Heat shock protein (HSP) 72 kD, a marker of cellular stress immunoreactivity in the spinal cord of morphine dependent rats on day 12 (MD12) and following 2nd day of morphine withdrawal (MWD2). Expression of HSP 72 kD is seen in the ventral (a) and lateral (b) horns in C-5 segment (arrows) on the day 12 of morphine dependent rats. The magnitude and intensity of HSP expression is further increased on  day 2 following morphine withdrawal in (MWD2, c,d, arrows). HSP expression is mainly seen in the cell cytoplasm. However, some nerve cells showed staining of cell nucleus as well. Bar = a.b = 40 µm, c,d = 30 µm [Reproduced with permission [12].

**Fig. (2):**
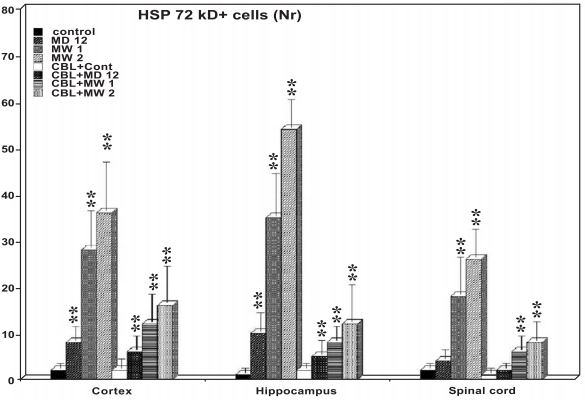
Semiquantitative analysis of HSP 72 kD expression in the CNS and its modification with cerebrolysin. The number of HSP positive cells significantly increased in the cerebral cortex, hippocampus and in spinal cord following morphine dependence by day 12 (MD 12) as well as after morphine withdrawal day 1 (MW1) and day 2 (MW2) as compared to control group. Cerebrolysin (CBL) given daily in control animals did not induce HSP expression (CBL+ Cont) but is able to significantly reduce the HSP expression in both morphine dependent (MD) and morphine withdrawal (MW) rats on day 1 and 2. ** = P < 0.01 from control group, Chi Square test.

**Fig. (3):**
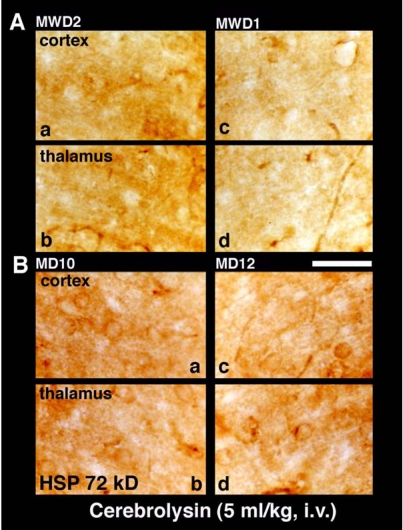
Light micrograph showing HSP expression in the cortex and thalamus is reduced by cerebrolysin treatment in morphine dependent rats on day 10 and 12 (MD 10, MD 12) and after morphine withdrawal day 1 and 2 (MWD1, MWD2). Only few scattered labeled neurons and dendrites particularly in the cell cytoplasm could be seen expressing weak HSP activity in cerebrolysin treated morphine dependent rats that was most prominent on day 10 (MD 10) as compared to day 12 (MD 12). On the other hand, cerebrolysin was able to prevent HSP expression in cortex and thalamus after morphine withdrawals day 1 and 2. Bar = 40 µm.

Cerebrolysin treatment markedly attenuated morphine dependence and/or withdrawal induced HSP 72 kD expression in the brain and spinal cord (Figs. **1**-**3**). Normal rats did not show HSP expression in the CNS [11, 12]. However, HSP expression was seen markedly upregulated on day 12 of morphine dependence as compared to the control group (Fig. **1**). This expression of HSP was further enhanced following morphine withdrawal in different brain regions of the rats (Figs. **1** and **2**).  This overexpression of HSP was most pronounced on day 2 of morphine withdrawal (Fig. **2**). Cerebrolysin treatment markedly attenuated the HSP expression seen on the morphine dependent rats on day 12 (Fig. **2** and **3**). Furthermore, cerebrolysin was also able to effectively reduce the expression of HSP in several brain and spinal cord regions following morphine withdrawal on day 1and 2 (Figs. **2** and **3**).

### Effect of Cerebrolysin on Morphine Induced Ultrastructural Changes

**Fig. (4):**
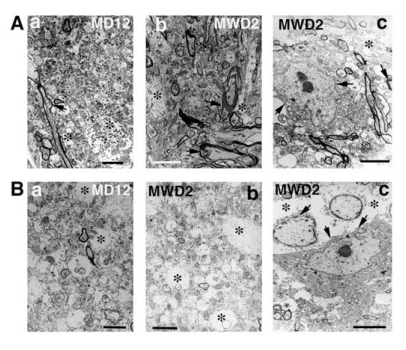
Ultrastructural changes in the nerve cell, myelin and neuropil in morphine dependent rats (day 12, MD 12) and following 2nd day of withdrawal (MWD2). A. Vacuolation (*), vesiculation of myelin (arrow) and degenerative changes in the neuropil are common in morphine dependent rat on day 12 (a). The magnitude and intensity of these structural changes, e.g., myelin vesiculation (arrows), membrane damage and vacuolation (') are much more frequent in animals following 2nd day of morphine withdrawal (A.b). One nerve cell showing dark and  condensed cell cytoplasm in the ventral medial thalamic nucleus (c) is clearly seen on the 2nd day of morphine withdrawal in the rat. The surrounding neuropil showed many degenerative changes (arrows). B. Vacuolation and degenerative changes in the piriform cortex in one morphine dependent rat on day 12 (MD 12, B.a). These degenerative changes are much more prominent in animals on the 2nd day of morphine withdrawal in the cortex (B.b) and in cerebellum (B.c). In the cerebellum, one Purkinje cell showed condensed cytoplasm with marked degenerative changes in the surroundings. Vacuolation (*) and degenerative changes are clearly seen around the nerve cell and the granule cell or astrocyte (MWD2 c). Bar: A.a = 1 µm, b,c = 0.8 µm; B.a,b = 1.5 µm, c = 0.8 µm [Reproduced with permission [12].

Cerebrolysin was able to reduce ultrastructural changes in the neuropil following morphine dependence or withdrawal in rats as seen using transmission electron microscopy (TEM). In untreated rats, morphine dependent rats on the day 12 showed vacuolation in the neuropil with shrunken cells and vesiculation of myelin (Fig. **4**). In this group, shrunken neurons and damaged synapses, axonal swellings are very common (Fig. **4**). Membrane disruption and edema formation are also frequent in various brain and spinal cord in these morphine dependent rats (results not shown). These neuronal, axonal and synaptic changes were further enhanced following morphine withdrawal (Fig. **4**). These ultrastructural damages were most marked on day 2 of morphine withdrawal (Fig. **4**). Accordingly, neuronal damage with distorted nucleus and nucleolus is quite frequent in rats in this group (Fig. **4**). Damage of perineuronal glial cells and microglia are also very common and destruction to neuropil, membrane disruption and vacuolation of myelin are most frequent in these rats on the 2^nd^  day of morphine withdrawal (Fig. **4**). These ultrastructural neurophil changes either caused by morphine dependence or withdrawal (results not shown) were considerably reduced by cerebrolysin. Thus, in cerebrolysin treated animals the neuronal, axonal and myelin structures were better preserved than untreated rats following morphine dependence either on the day 12 or on the 2^nd^ day after morphine withdrawal as compared to untreated group.

### Effect of Cerebrolysin on Morphine Induced La^3+^ Extravasation

**Fig. (5):**
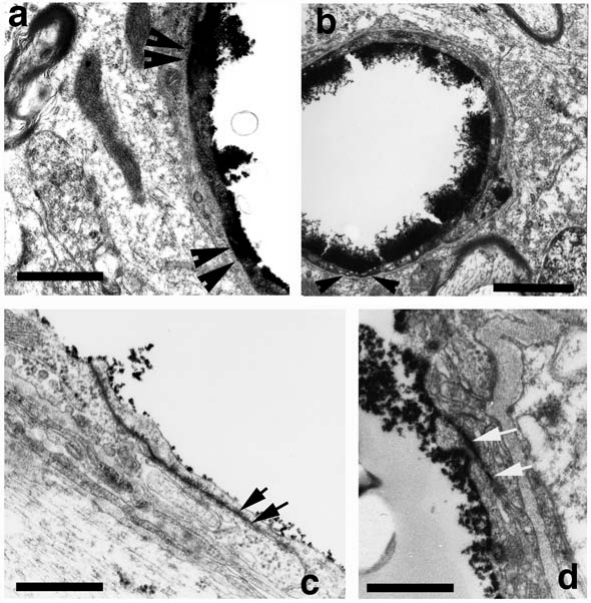
Representative examples of lanthanum extravasation across the blood-brain (a) and blood-spinal cord (b) barriers following 48 h after spontaneous morphine withdrawal in dependent rat. Infiltration of lanthanum is seen (arrowheads) across the endothelial cell of one microvessel from the cerebral cortex (a) and from the cervical spinal cord (b). Occurrences of many microvesicular profiles are clearly evident in the spinal cord endothelial cells (b). The tight junctions appear to be closed for lanthanum (arrows) in the cerebral (c) and spinal cord (d) microvessels. Edematous swelling of perivascular astrocytes is clearly visible (a-d). Bar = 500 nm (a); 600 nm (b), 300 nm (c); 400 nm (d).

**Fig. (6):**
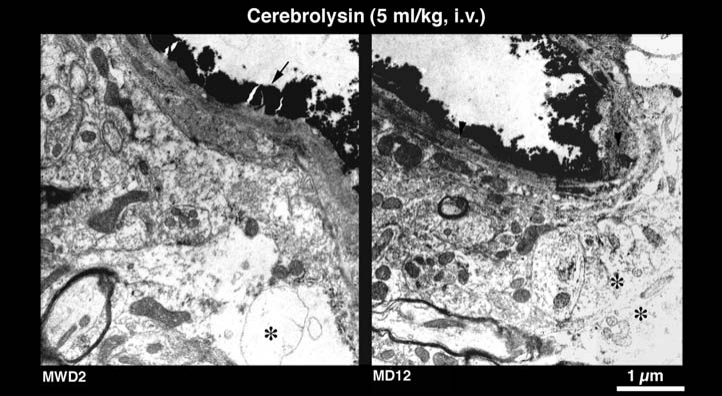
Effect of Cerebrolysin on lanthanum extravasation across the cerebral microvessels in morphine dependent animals on day 12 (MD12) and following 2^nd^ day of morphine withdrawal (MWD2). At the ultrastructural level, cerebrolysin was able to thwart lanthanum extravasation from lumen to the endothelial cell cytoplasm or in neuropil in MWD2 rat although; mild perivascular edema is also seen around this microvessel (*). Whereas, extravasation of lanthanum across the neuropil was largely abolished except in some very minor areas of the endothelial cell membrane (arrow head) in morphine dependent animal on the day 12(MD12). Signs of perivascular edema and structural changes are considerably reduced by cerebrolysin treatment.

Cerebrolysin was able to markedly reduce infiltration of lanthanum across the brain and spinal cord microvessels caused by morphine dependence and or morphine withdrawal (Table **3**). In normal animals, lanthanum is confined within the capillary lumen [50] and the endothelial cells and basal lamina do not show any infiltration of the ionic tracer within the neuropil [12, 51, 52]. However, morphine dependence results in infiltration of the electron dense lanthanum tracer within the endothelial cell cytoplasm (Fig. **5**) and even at the tight junctions that are normally closed (Fig. **5**). On the other hand, morphine withdrawal induces widespread leakage of lanthanum within the endothelial cells and the tracer could also be seen frequently in the basal lamina and within the neuropil (Fig. **5**). These effects of morphine withdrawal on the lanthanum exudation in the brain were most prominent on the 2^nd^ day after withdrawal (Fig. **5**).  Treatment with cerebrolysin markedly attenuated the exudation of lanthanum across the microvessels in morphine dependent rats or following morphine withdrawal. The most marked effects of cerebrolysin in reducing lanthanum extravasation across the microvessel were observed on the 2^nd^ day of morphine withdrawal (Fig. **6**). The incidence of perivascular edema in these groups was also reduced considerably by cerebrolysin treatment (Fig. **6**).

## DISCUSSION

The salient new findings of this investigation clearly show that cerebrolysin, a mixture of various neurotrophic factors and peptides is able to attenuate morphine dependence or withdrawal induced HSP response and neurotoxicity. This suggests that cerebrolysin could be used for the treatment of drugs of abuse related CNS dysfunction, not reported earlier.  Heat shock proteins (HSPs) are commonly known as stress proteins and are present in almost all neuronal and non-neural cells in any organism [49, 53]. Their expression denotes largely cellular activation or cellular stress [48-50]. Over-expression of HSPs in the CNS could be seen either following trauma, hyperthermia, hypoxia, and stressful situations or following exposure to psychostimulants, such as cocaine and methamphetamine [7-16]. It is believed that psychostimulant induced abnormal expression of HSP could reflect the state of specific cellular stress in the CNS. However, the functional significance of such findings is still not well known [9, 11-13, 45].

Our laboratory has initiated a series of investigations on the functional significance of HSP expression in the CNS in various animal models of stress, trauma and exposure to drugs of abuse in relation to cell injury or repair [45-50]. We hypothesize that upregulation of inducible types of HSP expression (HSP 72 kD in the CNS represent profound cellular injury, stress or over activation of neuronal and non-neuronal cells [7, 35, 48-50, 53]. Thus, attenuation of HSP 72 kD response using drugs or neurotrophic factors could lead to neuroprotection [11, 12, 46-50]. A reduction in HSP expression in morphine dependence and withdrawal by cerebrolysin further supports this hypothesis.  Previous reports from our laboratory show that morphine withdrawal and methamphetamine administration induces HSP 72 kD immunoreactivity in some brain areas. These brain regions are often associated with leakage of the serum albumin, a sign of the blood-brain barrier (BBB) disruption [7-13, 35, 45]. Thus, it is quite likely that disruption of the BBB caused by morphine and methamphetamine may lead to upregulation of HSP expression.

The present investigation further show that morphine withdrawal or dependence are associated with profound behavioral changes [11, 12], and cellular stress within the CNS as seen by marked overexpression of HSP 72 kD immunoreactivity. Interestingly, the brain areas of HSP expression in morphine dependence exhibited profound cellular damages that was also seen at the ultrastructural level. Although, during morphine dependent phase there is a reduction in the BBB permeability to proteins [11, 12], the cell changes and HSP expression is clearly seen in the brain of morphine dependent animals. This suggests that abnormality of BBB dysfunction; either increase or decrease in nature is associated with brain damage [14-16, 54].  This hypothesis is further confirmed in this investigation using electron microscopy. The ultrastructural observations showed shrinkage of neurons and non-neural cells during morphine dependence. This indicates that a decrease in the BBB function to radioiodine [11, 12] during morphine dependence is also associated with cellular abnormalities in the brain [54]. This is further corroborated by our investigation of endothelial cells at the ultrastructural level using lanthanum tracer. Thus, in morphine dependent animals, infiltration of lanthanum was largely limited to the luminal endothelial cell membrane and cell cytoplasm. The abluminal cell membrane and tight junctions appears to be largely intact. That is why lanthanum ion is not seen in the adjacent neuropil. This indicates that during morphine dependence, radiotracers are largely confined within the endothelial cells cytoplasm due to a defect in the luminal cell membrane permeability and could not enter into the brain compartment due to intact abluminal cell membrane function. Thus, it appears that morphine dependence could be able to affect large luminal endothelial cell membrane permeability [38]. However, this is a new feature that requires additional investigations. 

When cell membrane function of the endothelial cells is compromised it could lead to various ionic, immunological, biochemical or molecular alterations in the adjacent neuropil. Thus, cellular damage seen in such situations could be associated with cellular and/or molecular stress [12, 53]. This observation suggests that intoxication with drug like morphine could lead to profound cellular stress in the CNS through altering cell membrane functions leading to neurotoxicity and neurodegenerative changes in the brain over time. These observations are in line with the idea that alterations in endothelial cell membrane function could lead to cell damage.  Moreover, it is interesting to note that during morphine dependent phase no apparent abnormalities or alterations in sensory motor functions or in cognitive behavior may be noted in these animals. The signs of external stress reactions are largely absent in these morphine dependent animals, but their brains and spinal cords exhibit pronounced HSP expression and cell injuries. This suggests that drugs of abuse, e.g., morphine, cocaine, methamphetamine or ecstasy (MDMA) could induce profound brain damage without showing any apparent signs of behavioral dysfunction. These findings suggest that cellular stress or tissue damage in the CNS could be unrelated to their narcotic activities. Our observations further show that cerebrolysin treatment markedly attenuated cell damage and HSP expression in morphine dependent animals. This indicates that exogenous supplement of neurotrophic factors in combination, e.g. cerebrolysin could reduce cellular stress and brain damage during drug dependence. 

Another important new finding of this investigation is that cerebrolysin is able to reduce stress symptoms markedly following morphine withdrawal. This suggests that cerebrolysin supplement could also reduce withdrawal symptoms of abused drugs, e.g., morphine. This idea is further supported by the fact that cerebrolysin treatment during morphine withdrawal prevented HSP activation in the brain and spinal cord. Obviously, if stress signals are not reaching the brain and spinal cord following morphine withdrawal, the cellular stress and concomitant increase in HSP 72 kD will be reduced considerably [46-50].  The mechanisms by which cerebrolysin is able to reduce morphine withdrawal symptoms and HSP expression is unclear from this investigation. However, available evidences suggest that morphine withdrawal alter neurotrophic receptors activation and could also depletes stores of endogenous neurotrophins in the CNS [28, 55-58, 61]. A reduction in cell and tissue stores of neurotrophins is likely to enhance the vulnerability of cellular injury to external stimuli [61-63]. This is clearly evident by the neuronal damage and breakdown of the endothelial cell membrane permeability to lanthanum during morphine withdrawal. Thus, exogenous supplement of cerebrolysin during morphine withdrawal is able to reduce cell and tissue injuries and prevented HSP activation [35]. It is likely that exogenous administration of cerebrolysin could replace at least partially the cellular stores of neurotrophins in the brain during morphine withdrawal resulting in alleviating stress signals, cell injury and downregulation of HSP expression.

A reduction in HSP expression during morphine dependence or withdrawal by cerebrolysin appears to be neuro-protective in nature [45-50, 61-63]. Thus, cerebrolysin treatment not only reduced HSPs expression in the brain and spinal cord after morphine dependence or withdrawal, but also reduced the neuronal, glial and endothelial cell injuries. This suggests that overactivation of inducible type of HSP (72 kD) represent adverse life threatening injury signals [48-50]. This is supported by our observations that HSP expression is seen in the areas showing neuronal distortion, vacuolation or exhibiting edematous changes in the neuropil. Cerebrolysin treatment attenuated HSP expression and also resulted in marked neuroprotection. We speculated that the expression of inducible isoform of HSP 72 kD that is normally not present in the cells when activated by any adverse stimuli may represent cellular overactivation or cellular stress reaction [48, 50]. However, HSP 70 and other isoforms of stress proteins, e.g., HSP 27, HSP 90 that are normally expressed constitutively in cells and tissues could be neuroprotective in nature when they are upregulated following stress or injury signals [46-50, 61-63].

Previous reports from our laboratory also show that exogenous supplement of brain derived neurotrophic factors (BDNF) in spinal cord injury prevents the overexpression of trauma induced HSP 72 kD activity and subsequently reduces microvascular permeability disturbances, edema formation and cell damage [35-38, 45-50, 61-63]. This neuroprotective activity of BDNF in spinal cord injury was further strengthened when a combination of BDNF and glial cell line derived neurotrophic factor (GDNF) was given in combination [35-38, 61-65]. These observations suggest that during CNS injuries an exogenous supplement of both the brain and glial derived neurotrophic factors are needed to achieve some degree of neuroprotection. The present study further confirms this hypothesis and clearly indicates that cerebrolysin that represents a combination of neurotrophic factors from both the neurons and glial cells has profound neuroprotective efficacy in drug dependence and withdrawal induced brain damage.

Taken together, our observations are the first to show that cerebrolysin is able to attenuate cell injury in morphine dependence and withdrawal and thus could be an important therapeutic tool to achieve neuroprotection in morphine and other psychostimulant induced neurotoxicity. This neuroprotection caused by cerebrolysin following morphine dependence and withdrawal is achieved by reducing cellular stress in the CNS as evident with downregulation of HSP activity.  In conclusion, our observations point out a new role of cerebrolysin for the treatment of drugs of abuse induced disorders in clinical situations. However, further research is needed to find out a clinical role of cerebrolysin in patients affected by drugs of abuse syndrome.

## Figures and Tables

**Table 1:** Effect of Cerebrolysin on Stress Symptoms During Morphine Dependence and Withdrawal

Parameters	Control	Morphine Dependence			Morphine Withdrawal			
		1st Day MD1	10th Day MD10	12th Day MD12	12 h MWD0.5	24h MWD1	48h MWD2	72h MWD3
**I. Stress symptoms**	n=6	n=6	n=8	n=12	n=8	n=14	n=16	n=8
*A. Untreated*								
Wet-Shakes	nil	nil	nil	nil	4±2	8±3^a^	6±2	5±3
Piloerection	nil	nil	nil	nil	+++	+++++	++++	+++
Writhing	nil	nil	nil	nil	+++	+++++	++++	+++
Teeth chattering	nil	nil	nil	nil	+++	+++++	+++++	+++
Diarrhoea	nil	nil	nil	nil	+++	+++++	+++	nil
Aggressive behaviour	nil	nil	?	?	+++	+++++	+++++	+++++
Microhaemorrhages in stomach	nil	6±5	8±5	12±8	48±12	68±18#	85±14#	23±8#
*B. Cerebrolysin treated 5 ml/kg, i.v.*								
Wet-Shakes	nil	nil	nil	nil	4±2	4±2*	3±2*	3±2*
Piloerection	nil	nil	nil	nil	+++	+++++	++++	+++
Writhing	nil	nil	nil	nil	+++	+++++	++++	+++
Teeth chattering	nil	nil	nil	nil	+++	+++++	+++++	+++
Diarrhoea	nil	nil	nil	nil	+++	+++++	+++	nil
Aggressive behaviour	nil	nil	?	?	+++	+++++	+++++	+++++
Microhaemorrhages in stomach	nil	nil	2±1*	2±3*	6±2*	12±4*	16±8*	12±4*

values are mean±SD;

# = Many microhaemorrhages;

a = significantly different (P<0.05) from Morphine withdrawal 12 h;

+++ = mild

++++ = moderate

+++++ = severe

? = unclear, nil = absent

MD = morphine dependent; MWD = Morphine withdrawal;

* = P<0.05, Chi Square test from untreated group;

a = P<0.05, compared from MD12 group; For details see text.

**Table 2:** Effect of Cerebrolysin on Physiological Variables During Morphine Dependence and Withdrawal

Parameters	Control	Morphine Dependence			Morphine Withdrawal			
		1st Day MD1	10th Day MD10	12th Day MD12	12 h MWD0.5	24h MWD1	48h MWD2	72h MWD3
**II. Physiological variables**	n=6	n=8	n=8	n=6	n=8	n=8	n=6	n=6
*A. Untreated*								
MABP torr	110±8	122±8**	140±6**	148±5**	90±4**	128±6**	146±14**	94±8
Arterial pH	7.38±0.02	7.36±0.08	7.35±0.07	7.36±0.05	7.35±0.06	7.36±0.08	7.34±0.05	7.36±0.07
PaO2 torr	81.56±0.23	80.34±0.32	80.28±1.01	78.34±1.34	81.87±0.23	82.45±0.34**	82.67±0.31**	81.68±0.28
PaCO2 torr	34.62±0.34	33.32±0.22	33.54±0.14	33.10±0.43	33.21±0.43	31.54±1.24	32.06±1.34	32.34±0.98
Body Temp ° C	37.61±0.42	39.42±0.41**	38.64±0.51	38.42±0.31	39.54±0.23**	40.23±0.18**	39.28±0.11**	38.67±0.22
Body weight (g)	288±14	289±8	340±12**	356±8**	350±8*	340±6*	320±8**	310±12*
Heart rate beats/min	280±12	320±18*	330±12**	338±10**	304±8*	310±8*	308±7*	296±14
Respiration cycles/min	76±6	80±8	84±6**	86±7**	89±5*	94±4**	92±5**	80±8
*B. Cerebrolysin treated 5 ml/kg, i.v.*								
	n=6	n=6	n=7	n=6	n=6	n=7	n=8	n=8
MABP torr	108±4	112±6	128±8**	138±8**	96±8**	108±7	126±8*	104±9
Arterial pH	7.38±0.04	7.34±0.05	7.32±0.06	7.33±0.04	7.30±0.04	7.37±0.04	7.32±0.06	7.30±0.04
PaO2 torr	81.36±0.13	81.34±0.23	80.64±0.61	79.04±0.84*	81.07±0.63	81.40±0.14	81.37±0.51	81.08±0.34
PaCO2 torr	34.35±0.22	34.06±0.18	33.84±0.24	33.80±0.63	33.62±0.83	32.14±0.24	32.16±0.24	32.54±0.32
Body Temp ° C	37.44±0.36	38.64±0.21*	38.34±0.41*	38.32±0.11*	39.44±0.13**	40.03±0.28*	39.48±0.31*	39.07±0.44
Body weight (g)	290±12	294±10	328±10*	340±10**	336±12**	330±8**	326±10*	320±8**
Heart rate beats/min	288±14	310±12*	320±8**	328±8**	318±6*	300±5*	288±10	286±8
Respiration cycles/min	72±8	76±4	80±4**	88±6**	80±8*	84±6*	80±5*	78±6

values are mean±SD; a = significantly different (P<0.05) from Morphine withdrawal 12 h; nil = absent,; MD = morphine dependent; MWD = Morphine withdrawal For details see text.

* P <0.05;

** P < 0.01, ANOVA followed by Dunnett's test for multiple group comparison from one control group.

**Table 3:** Semiquantitative Data on Lanthanum Extravasation in Vascular Profiles in the Brain Following Morphine Dependence or Withdrawal in Control and Cerebrolysin (5 ml/kg, i.v.) Treated Rats

Type of Experiment	n	Lanthanum Distribution in 80 Microvascular Profiles (Nr.)									
		Inside Lumen		Endothelial Cell Cytoplasm		Basal Lamina		Vesicular Profiles		Between the Tight Junctions#	
		Cerebellum	Cortex	Cerebellum	Cortex	Cerebellum	Cortex	Cerebellum	cortex	Cerebellum	Cortex
*A. Untreated*											
Control	5	76±4	78±2	0	0	0	0	6±4	8±5	0	0
Morphine 12th day	6	54±4*	52±8*	12±6*	8±6*	8±4*	6±4*	18±6*	12±3	1±1	1±2
MD12											
Morphine withdrawal 24 h	6	42±4*	48±6*	34±11*	26±14*	22±8*	16±7*	36±18*	28±14*	1±2	3±2
MWD1											
Morphine withdrawal 48 h	5	34±12*	42±10*	48±8*	36±7*	46±14*	33±18*	44±12*	38±14*	2±2	2±3
MWD2											
*B. Cerebrolysin treated*											
Control	5	78±2	78±5	0	0	0	0	0	8±4	0	0
Morphine 12th day	6	14±6*	12±4*	4±2*	0	0	0*	0*	5±4	2±1	1±1
MD12											
Morphine withdrawal 24 h	6	12±8*	18±4*	8±2*	2±1*	3±2*	1±1*	3±2*	3±2*	2±2	1±2
MWD1											
Morphine withdrawal 48 h	5	8±6*	12±6*	10±4*	4±2*	4±3*	2±1*	4±2*	4±2*	2±3	2±2
MWD2											

Data from 6 to 8 animals in each group; values are mean±SD;

* = P <0.01, Chi-square test, Significantly different from control group,

# = lanthanum is seen between the two tight junctions and stopped at the second one.
